# Towards an ultra-rapid smartphone- connected test for infectious diseases

**DOI:** 10.1038/s41598-017-11887-6

**Published:** 2017-09-20

**Authors:** Valérian Turbé, Eleanor R. Gray, Victoria E. Lawson, Eleni Nastouli, Jennifer C. Brookes, Robin A. Weiss, Deenan Pillay, Vincent C. Emery, C. Theo Verrips, Hiromi Yatsuda, Dale Athey, Rachel A. McKendry

**Affiliations:** 1 London Centre for Nanotechnology, Faculty of Maths and Physical Sciences, University College London, 17–19, Gordon Street, London, WC1H 0AH UK; 2 Division of Infection and Immunity, University College London, London, WC1E 6BT UK; 3 OJ-Bio, International Centre for Life, Times Square, Newcastle-upon-Tyne, NE1 4EP UK; 4 Department of Clinical Virology, University College London NHS Foundation Trust, London, W1T 4EU UK; 5 Department of Population, Policy and Practice, UCL GOS Institute of Child Health, 30, Guilford St, London, WC1N 1EH UK; 6 Africa Health Research Institute, KwaZulu Natal, South Africa; 7 Department of Microbial and Cellular Sciences, University of Surrey, Guildford, Surrey, GU2 7HE UK; 8 QVQ Holding B.V., Utrecht, The Netherlands; 9 Japan Radio Co. Ltd., Saitama, 356-8510 Japan; 10 Division of Medicine, University College London, London, WC1E 6BT UK

## Abstract

The development is reported of an ultra-rapid, point-of-care diagnostic device which harnesses surface acoustic wave (SAW) biochips, to detect HIV in a finger prick of blood within 10 seconds (sample-in-result-out). The disposable quartz biochip, based on microelectronic components found in every consumer smartphone, is extremely fast because no complex labelling, amplification or wash steps are needed. A pocket-sized control box reads out the SAW signal and displays results electronically. High analytical sensitivity and specificity are found with model and real patient blood samples. The findings presented here open up the potential of consumer electronics to cut lengthy test waiting times, giving patients on the spot access to potentially life-saving treatment and supporting more timely public health interventions to prevent disease transmission.

## Introduction

Ebola and Zika viruses offer a stark reminder that infectious diseases rank among the gravest threats to human health, and can spread rapidly and unpredictably. New infections will continue to emerge each year, and old enemies re-emerge, increasingly with acquired-drug resistance (e.g. gonorrhoea and HIV). Rapid diagnosis plays a crucial role in any outbreak situation, empowering patients to gain faster access to potentially life-saving treatment, and informing prevention strategies to protect the wider public. However, routine diagnostic tests based on enzyme linked immunosorbent assays (ELISAs) and polymerase chain reaction (PCR) are confined to centralized laboratories often requiring large, sophisticated, costly instrumentation and highly trained staff. Inherent delays occur between taking samples, conveying them to the laboratory, waiting for results to come back and subsequent follow up appointments^1–3^. This means that a patient often has to make multiple visits to a clinic in order to receive treatment, potentially over long distances. This delays prescribing of treatment with increased risk of suffering, mortality, and also incorrect prescription of antimicrobials.

Recent policy drivers aim to widen access to testing using so called ‘rapid’ point-of-care tests (POCT) but the performance and implementation of these tests still remain a challenge^4^. The most common tests based on lateral flow technology are still relatively slow, requiring a 10–20 minute waiting time for results^5^. This exceeds a typical doctor’s appointment (8–10 mins in the UK^6^) necessitating changes to patient pathways within a clinic with additional on-costs and staffing implications. It is also notoriously difficult to interpret a faint lateral flow test line by eye, particularly for non-experts (e.g. self-testers)^1^. Those tests that are currently available are insensitive to recent (acute) infections^7^ and lack the ability to automatically capture test results electronically, risking an incorrect reading, missed opportunities to link patients to care pathways and potential data loss for public health (e.g. during an Ebola outbreak)^8^. Alternatively, uneccesary treatment may be initiatied for those with false positive results^9^. An additional review of more recent diagnostics that have appeared in the literature (but not yet in the clinic) is given by Wang *et al*.^10^.

Here we report the development of an ASSURED (Affordable, Sensitive, Specific, User friendly, Rapid and Robust, Equipment Free and Delivered^11^) smartphone-connected SAW medical device, to detect biomarkers of HIV infection in a patient sample within seconds. The marriage between diagnostics and consumer electronics opens up the potential to dramatically widen access to testing and support convenient on the spot follow-up testing and care. With more mobile phone subscriptions than people on the planet (7.4 billion in 2016^12^), their reach could help to bring tests outside of centralized hospital laboratories, to where they are most needed at the point-of-care, particularly in resource-limited settings across the developing world and even into the home. Smartphone usage is on the rise with an estimated 2 billion subscriptions worldwide and rapid growth in developing countries −34% of South Africans currently own a smartphone and forecasts predict 500 million smartphones in Sub-Saharan Africa by 2020^13^. Beyond improving access, their battery life, processing power, display screen and inbuilt sensors could, in principle, be harnessed to significantly improve the performance of current POCTs and offer faster access to care, including follow up appointments in local clinics.

There have been a number of advances recently in the field of smartphone-connected diagnostics, both with *in vivo* and *in vitro* testing, using either the built-in smartphone sensors or their data transmission capabilities to link with existing diagnostic systems^14^. They include a smartphone-based system to monitor changes in pH in sweat or saliva, using disposable strips;^15^ colorimetric rapid quantification of vitamin D levels, using the built-in smartphone camera, paper strips and image analysis algorithms;^16^ as well as a more general smartphone-based reader for lateral flow immunochromatic assays^17^. Another recent development is the report of nanophotonic-devices which interface with smartphone cameras to read out current commercial lateral flow tests^18^, overcoming the need to visually read tests by eye. Microfluidic technologies such as the mChip and a ‘dongle’ recreate all the functions of an ELISA to diagnose HIV and syphilis within 15 minutes with nanoparticle signal enhancements and 6 wash steps^19^. By contrast, our approach requires no optics, microfluidics, analyte labelling, amplification or wash steps and instead exploits tiny microelectromechanical piezoelectric SAW sensors. SAW microelectronic filters, found within every smartphone, are thus transformed into biochips which can directly detect disease biomarkers in bodily fluids such as blood.

This platform technology can in principle be applied to a range of diseases and here we focus on HIV as an exemplar. This choice was driven by the compelling unmet human and economic needs which have triggered major policy drivers to widen access to HIV testing to hospital emergency services, doctors’ surgeries, community outreach centers^20^ and self-testing in the home^21^. The AIDS pandemic ranks among the most devastating infectious diseases in human history, infecting more than 78 million people and resulting in 39 million deaths^22^ with tremendous burden of illness in Sub-Saharan Africa. A significant proportion of those infected remain unaware of their infection – estimates range from 17% in UK^23^ to 55% in sub-Saharan Africa^24^. Late diagnosis is associated with a 10-fold increased risk of death^23^ and increased risk of unknowingly transmitting the infection^25^. Early diagnosis and access to antiretroviral treatment increases life-expectancy by 10 years^26^, reduce infant mortality by 76%^27^ and in pregnant women can reduce the risk of transmitting the virus to their babies to less than 1%^28^. According to the US Centre for Disease Control and prevention (CDC), every case of HIV that is prevented saves $380,000 in lifetime treatment costs^28^.

We sought to engineer the first prototype smartphone-connected SAW device to diagnose HIV with high sensitivity and specificity, and to harness the processing power of smartphones to speed up the delivery of results to make testing more convenient and avoid lengthy waiting times. We first optimized SAW biochips and capture coatings to detect model HIV antibodies and recombinant antigens (anti-p24 and p24 respectively). This work was then extended to a proof of concept assay testing real patient samples, using differential measurements with reference tests to achieve high specificity and sensitivity within seconds.

## Results

### SAW biosensor device and biochips for HIV

**Figure 1:**
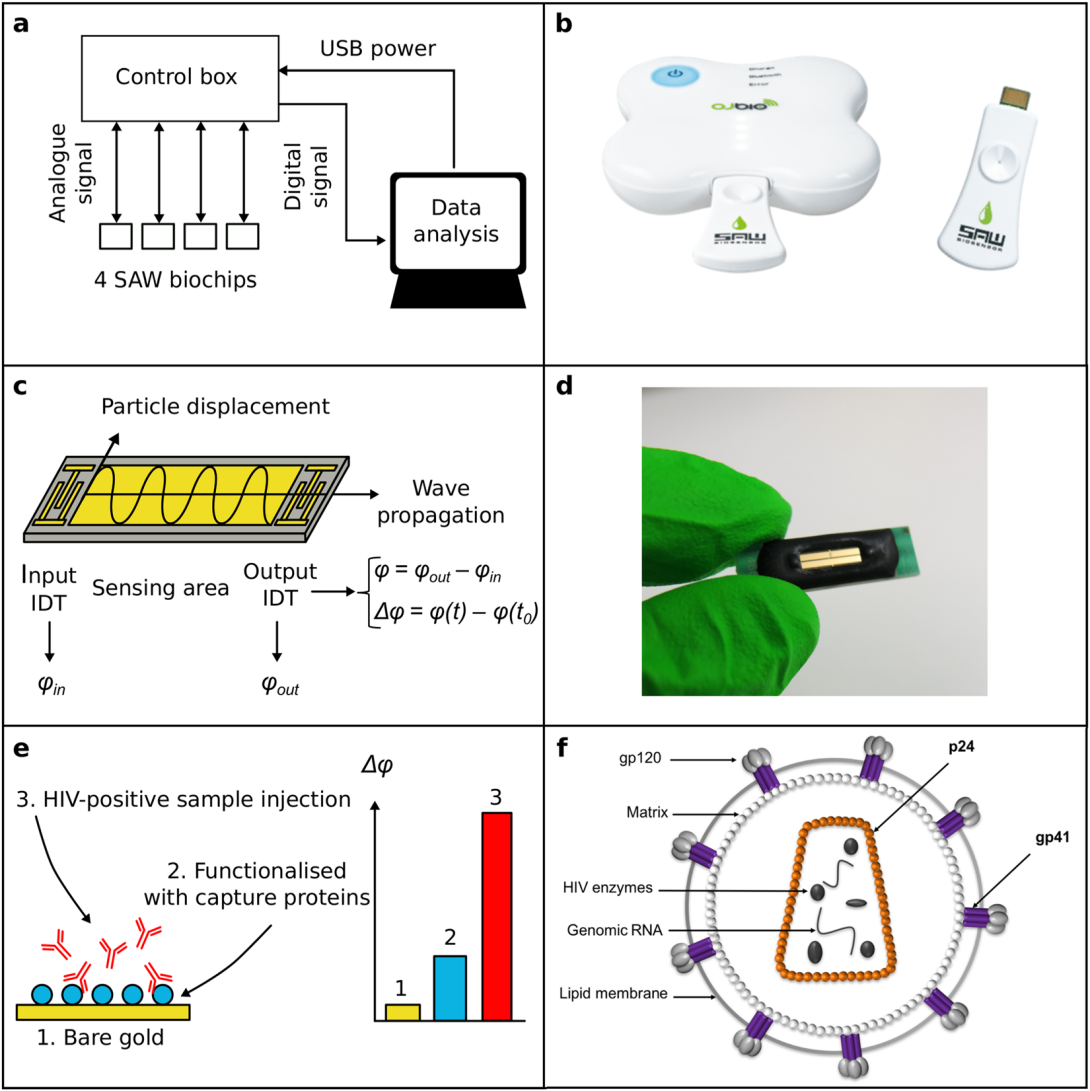
Smartphone-connected SAW test for HIV (**a**) Schematic to illustrate the laboratory prototype. The control box sends/receives an analogue signal to/from 4 SAW biochips in parallel, and transmits a digital signal to the smartphone (or laptop). An app (or software) processes and analyses the data. (**b**) Photograph of the hand held SAW development prototype. This figure is not covered by the CC BY licence. [Credits to H Yatsuda of Japan Radio Company]. All rights reserved, used with permission. (**c**) A schematic to illustrate the principle of SAW generation on biochips via the piezoelectric effect: The SH-SAW is transmitted from the input interdigitated electrode (left IDT) to the output IDT (right) and propagates along the sensing area. The relative phase shift *Δφ* measured between the time *t* and the start of the measurement *t*_*0*_ is continually measured to provide real-time analysis. (**d**) Photograph of a disposable SAW biochip measuring 25 mm × 7 mm × 2 mm. (**e**) A schematic to illustrate the concept of biosensing on SAW biochips: The sensing area (comprising a gold thin film, titanium adhesion layer on quartz) is functionalized with a monolayer of capture ligands (e.g. a protein that binds to the biomarker of interest, blue) using alkanethiol linker chemistry. A sample containing a HIV antibody biomarker (red) is shown in solution binding to the capture ligand. The resultant wave phase shift, *Δφ*, increases with the amount of capture protein bound to the surface and biomarker specifically bound to the biochip (3). (**f**) Schematic of HIV. Anti- p24 antibodies are raised against the viral protein p24, which forms the capsid of the virus (shown in orange). These antibodies are raised within two to three months at levels of 10–1000 µg/ml^31,32^. However, seroconversion can take up to six months^57^ and therefore recently acquired infections can pass undetected by these tests. During this window, the p24 capsid protein becomes detectable (2 to 3 weeks^58^).

The principle of SAW was first described by Lord Rayleigh in 1885^29^. Here we harness advances in microfabrication and the piezoelectric effect to generate a shear horizontal surface acoustic wave (SH-SAW) on a millimetre sized biochip. The wave generates a surface particle displacement perpendicular to the direction of the wave propagation^30^, and makes the biochip sensitive to reactions occurring on the surface. Our prototype devices and biochips are shown in Fig. 1(a–d) and described fully in the Experimental Section. In brief, our device comprises a disposable biochip, a pocket-sized control box reader and a mobile device (laptop or smartphone) to analyse, display and transmit results. Each biochip comprises a plane piezoelectric quartz crystal (36°Y-cut 90°X-propagation), with gold input and output interdigitated electrodes and a ‘sensing’ area in between. We tailored the sensing area with a thin gold film and a layer of capture proteins. If a biomarker of HIV is present in a finger prick of blood, it binds to the capture proteins on the surface of the biochip. This gives rise to a perturbation of mass and viscoelasticity which can be readily detected by the phase change of the SH-SAW - the difference in wave phase measured in degrees (°) between the input and the output electrodes (*Δφ*, Fig. 1e). The diagnosis of HIV is based on the detection of an immunological (anti-p24 antibody) and/or virological (p24 antigen) biomarker (Fig. 1f) which become detectable in blood at relatively defined time points post exposure^31–33^. Our laboratory prototype device comprises a small hand-held control box (measuring 14 × 10 × 4 cm) and up to four individual disposable SAW biochips (each measuring 25 × 7 × 2 mm) which can be connected in parallel for multiplexed analysis of biomarkers and control measurements. A more advanced development prototype device, containing all the same elements as the laboratory prototype, is shown in Fig. 1b, where the SAW biochip is mounted on a disposable cassette resembling a USB stick and results sent to a smartphone app either via a cable or via bluetooth. We note that all the measurements presented herein were performed using the laboratory prototype presented in Fig. 1a.

Key technical breakthroughs contributing to the performance of our ASSURED device include (i) *Affordable* - mass manufacture of SAW biochips using low cost quartz/gold materials instead of more common and expensive materials (e.g. lithium tantalate). Billions of SAW filters are manufactured each month for the mobile phone industry. The price per SAW filter is currently less than a couple of tens of cents each. It is expected that the total cost of goods will be economically viable due to the ability to mass manufacture since they are purely based on microelectronics, standard thin film deposition of gold, and bulk production of bio-reagents, therefore making the cost of goods compatible with single sample disposable tests. The detection circuit of the control box is expected to cost less than $30 and we are also looking at stand-alone disposable formats. The SAW biosensor presented here is also inherently low power and the control box can be charged by a smartphone. (ii) *Sensitive* – it operates at an optimized frequency of 251.5 MHz, 1–2 orders of magnitude higher than a typical quartz crystal microbalance benchtop analyzer (1–30 MHz). The SH-SAW is highly sensitive to changes in mass and mechanical properties of materials close to the biochip surface and minimizes losses to bulk modes^30^. (iii) *Specific diagnosis* – is achieved via a multiplex array of 4 biochips which allows differential measurements using reference biochips to reduce the risk of non-specific signals (e.g. temperature and viscosity) leading to false positive results. (iv) *User friendly* – results are electronically displayed on the user friendly app/software (iOS or Android) interface (e.g. ‘Reactive/Yes/No’), overcoming problems with interpreting and capturing lateral flow tests results. Results can be encrypted and securely sent to a local healthcare system to receive a follow up appointment. (v) *Robust and Rapid* – SAW biochips are small, rugged and very stable^34^. The patented micro fabricated glass-polymeric capping layer protects the interdigitated electrodes^35^ meaning we do not require any sophisticated microfluidics to be interfaced with the device, which can be prone to clogging. The biochips are insensitive to environmental lighting conditions, which can affect the interpretation of a lateral flow test and other optical assays^36^. Importantly, tests can be performed ultra-rapidly via direct detection methods overcoming the need for multiple steps, amplification and wash steps used in microfluidic devices. (vi) *Equipment free* – removes the need for costly instrumentation by using the smartphone battery supply, processing power and display screen. (vii) *Delivered* – in future this technology could leverage on established diagnostics and telecommunications supply chains.

### Detection of anti-p24 antibodies at clinically relevant levels

**Figure 2:**
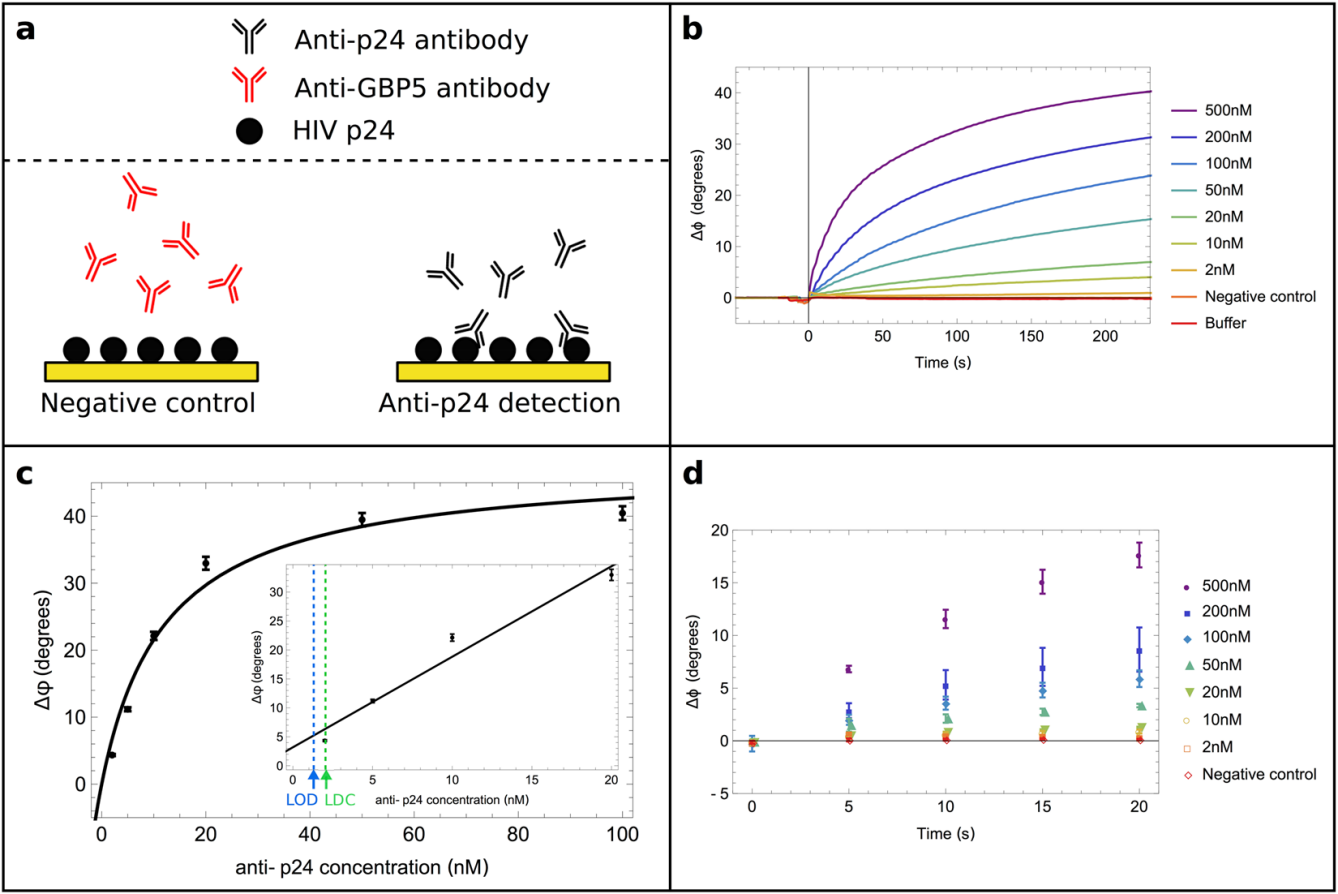
Anti-p24 antibody detection in buffer using a SAW biochip functionalised with HIV p24 recombinant proteins (**a**) Schematic illustrating the specific binding of anti-p24 HIV antibodies to p24 coated biochips, and a negative control using an antibody with no affinity for p24 (anti-GBP5 antibody) which shows no binding to the biochip. (**b**) Overlaid raw data plots to show the phase shift recorded between input and output IDTs as a function of time. The sample containing the anti-p24 antibody is injected at t = 0. Each sample trace was normalised with the reference assay (containing only buffer, orange line). (**c**) Graph showing the total phase shift recorded after 5 minutes, plotted as a function of anti-p24 antibody concentration. Each measurement was repeated 3 times; error bars shows the standard deviation of the mean. Black line: Langmuir adsorption isotherm of equation *y* *=* *47*.*8* (±*3*.*3*)*x*/(*1*2.2 (±*2*.*7*) + *x*), *R* ^*2*^ = *0*.*994* Inset: Zoom of linear regression in the range 2–50 nM, *y* = *1*.*56x* + *3*.*2*, *R* ^*2*^ = *0*.*964*. The estimated limit of detection (LOD) and the lowest detected concentration (LDC) are marked on the bottom axis by the blue and green arrows, respectively. (**d**) Plot showing the average phase shift recorded every 5 seconds during the first twenty seconds after injection. Each point represents the average of three measurements, errors bars represent the standard deviation from the mean. Samples of different concentrations (from 50 nM (7.5 µg/ml) and above) can be distinguished from one another 10 seconds after sample injection.

Anti-HIV antibodies are the most common target in POCT for HIV, the immunological response typically being detected several weeks after exposure to the virus. Here we initially focused on the detection of antibodies against the viral capsid p24, one of the most conserved and abundant proteins found in HIV. This is achieved using SAW biochips functionalised with recombinant p24 capture ligands (Fig. 2a) via dithiobis[succinimidyl propionate] (DSP) thiol linker chemistry, whereby the gold-sulphur bond drives the formation of a relatively well defined self-assembled monolayer and the terminal reactive ester group forms a covalent bond to the lysine residues on p24. We incubated a 20 µl drop of buffer for 2.5 minutes, to establish a stable baseline before loading the sample. The subsequent injection of 500 nM (75 µg/ml) anti-p24 antibodies triggered a rapid increase in the measured phase shift (*Δφ*) leading to a total phase shift of 46° after just five minutes (Fig. 2b). This phase shift indicates a change in mass and/or viscosity at the surface proximity associated with the specific binding of anti-p24 antibodies to p24 capture proteins on SAW biochips. We sought to carefully evaluate the analytical specificity of the SAW biochips, via negative control experiments. This involved injecting non-specific proteins - including a blank injection of buffer containing bovine serum albumin and then a non-specific antibody called anti-GBP5, with no known affinity to HIV p24, under exactly the same reaction conditions and concentration as the specific antibody, anti-p24. These results are shown in Fig. 2b - an injection of buffer with BSA shows no change in phase shift (orange trace Fig. 2b) and a sample containing 500 nM (75 µg/ml) of anti-GBP5 shows a negligible response (negative control, red trace Fig. 2b).

Next we systematically tested the relationship between the phase shift *Δφ* and the concentration of anti-p24 antibodies in solution at clinically relevant levels and found it scaled to a Langmuir adsorption isotherm, R^2^ = 0.994 (Fig. 2c). All measurements were performed in triplicate. At low antibody concentrations (5–20 nM, which corresponds to 0.75–3 µg/ml), the phase signal increases linearly from 4 ± 0.1 to 22 ± 0.6°. Above 20 nM (3 µg/ml), the signal begins to plateau, presumably when all the available binding sites on the surface are occupied. Testing three independent biochips, the lowest detected concentration (LDC) was found to be 2 nM (300ng/ml) anti-p24 antibodies in buffer within 5 minutes and the limit of detection (LOD) is 1.1 nM (165ng/ml), estimated using the function fitting the data presented in the insert of Fig. 2c, and defined as the concentration corresponding to a signal readout of the mean plus three times the standard deviation of the measurements taken for 0 nM samples. Both the LDC and LOD are well below the clinically relevant range of concentrations for anti-p24 antibodies, which is reported to be 82–1,900 µg/ml, or 0.55–12.67µM^32^. The small error bars reflect the high reproducibility of these assays between different biochips and functionalisation steps. These results compared favourably to those obtained using commercial lateral flow tests which we have found can detect 10–30 nM (1.5–4.5 µg/ml).

### Testing times - ultra-fast diagnosis of HIV within seconds

Having established that SAW biosensors can detect antibodies at clinically relevant levels, we sought to test how quickly results could be delivered by analysis of the phase change in real-time. Reducing the time to deliver a test result is crucial for its utility within a typical primary healthcare setting, allowing treatment and care to be given on the spot, overcoming the need for multiple visits and reducing the risk that patients may not return to receive their results. The faster the test can be delivered the more time will be available for post-test counselling and care. The Foundation for Innovative Diagnostic (FIND)’s target product profile for a HIV self-test for use in the home underlines the need for results in under 5 minutes^37^.

Figure 2d shows the average phase shift (for three biochips) recorded shortly after sample injection, for the range of antibody concentrations tested. The results demonstrate that clinically relevant HIV antibody concentrations can be detected and distinguished just 10 seconds after injection of sample (sample-in-result-out) down to 50 nM (7.5 µg/ml). To our knowledge there are no tests commercially available or reported in the literature that are this rapid and exhibit a similar limit of detection; those currently on the market require a visual readout with the associated difficulties^1^.

### Detection of recent HIV infections using recombinant p24 antigen detection

Building on the detection of HIV antibodies on SAW biochips, we next sought to investigate whether SAW biochips could detect a virological marker of infection, namely p24 antigen. This is a key biomarker of acute HIV infections and is used in combination with antibody detection in ‘gold-standard’ fourth generation HIV diagnostic assays in centralised laboratories. However, this protein is only present in human blood in minuscule levels, around a million-fold lower concentrations than its antibody counterpart^32,38–42^ and a billion times lower than human serum albumin, presenting a major challenge for POCT diagnostics. Fourth generation p24 POCT are beginning to emerge but their performance in the field has been suboptimal, and this has been attributed to a number of factors, including the low concentration of p24 antigen in patient samples^4^.

Here we present the first proof of concept for detection of p24 by developing SAW biochips functionalised with novel capture ligands engineered from anti-p24 llama antibodies. These novel capture ligands are llama VHH (the Variable region of the heavy chain of heavy chain-only antibodies), and are one-tenth the size of conventional antibodies^43^. Their small footprint means that VHH can access hidden clefts which are inaccessible to larger proteins and in principle they can be packed into dense arrays of capture ligands for diagnostic applications. They have remarkable temperature stability^44^, have shown strong affinity to p24^45^ and are therefore ideal candidates for capture ligands on SAW biochips. We immobilised the llama VHH on SAW biochips using DSP chemistry. Characterisation by X-ray photoelectron spectroscopy provides evidence of monolayer coverage, which we estimate to be around 0.11 VHH/nm^2^ (Figure S1 in Supplementary Information).

**Figure 3:**
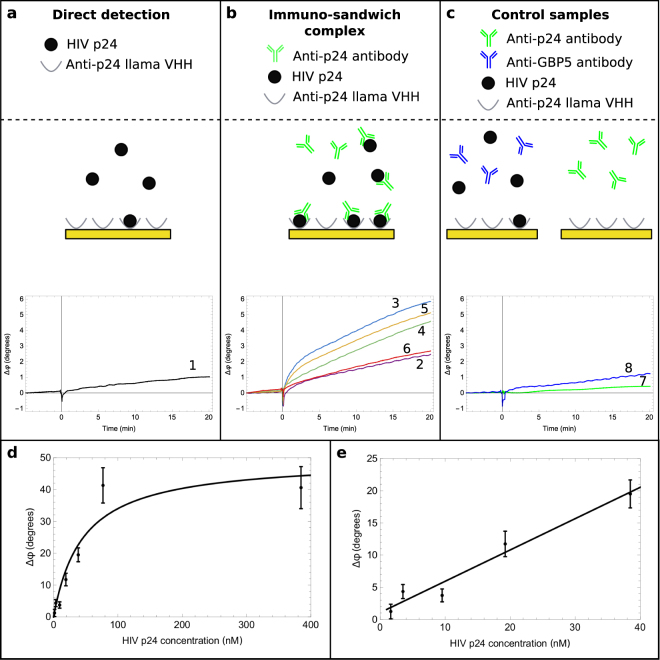
HIV p24 detection in buffer using a SAW biochip functionalised with anti-p24 llama VHH. Schematic showing the immuno-sandwich used to detect HIV p24 (the chip is coated with anti-p24 llama VHH) and the resultant phase shift recorded between the input and the output IDT as a function of time. The numbers shown on the graphs refer to different immuno-sandwich complexes formed using the anti-p24 antibodies listed in Table 1. Samples injected at t = 0. (**a**) Direct HIV-p24 detection (llama VHH capture + p24 only). (**b**) Immuno-sandwich p24/anti-p24 complexes are formed in the sample and bind to the functionalised surface. The largest signal is seen for immuno-complex 3. (**c**) Control samples, where no p24/anti-p24 complexes are formed. (**d**) Titration of HIV-p24 using optimised immuno-sandwich with NIH-3537 anti-p24 antibody (number 3 in Table 1). The phase shift was recorded 5 minutes after sample injection. Black line shows Langmuir isotherm fit of equation *y* *=* *49*.*6 (* ± *7*.*7)x / (45*.*8 (* ± *19*.*9)* + *x)*, *R* ^2^ = *0*.*959*. Data shown are the combined results from three measurements and error bars show standard deviation of the mean. (**e**) Zoom on the 0–40 nM region and fitted with a linear regression model (black line) of equation *y* = *0*.*49x* + *0*.*94*, *R* ^2^ = *0*.*964*. Data shown are the combined results from three measurements and error bars show standard deviation of the mean.

Figure 3a shows direct p24 detection on SAW biochips coated with anti-p24 llama VHH. A sample concentration of 4 nM (96 ng/ml) p24 gave rise to a phase change of only 1.2°, even after a 20 minute incubation. Since p24 is only one-sixth the mass of an antibody, a strategy of signal amplification was required through the creation of a larger immuno-sandwich, to achieve detection of p24, corresponding to a six-fold increase in mass per antigen. We tested five combinations of detection antibodies from commercial sources and the AIDS Reagent Program. All antibodies used in Fig. 3b are described in Table 1.

**Table 1:** Samples used for HIV p24 detection assay optimisation.

Sample	HIV p24	Detection antibody	Binding to HIV p24
1 (control)	Yes	—	—
2	Yes	BC1071 (Aalto Bio Reagents,UK)	Yes
3	Yes	NIH-3537 (AIDS Reagent Program, USA)^46^	Yes
4	Yes	NBS500–473 (Novus Biologicals, UK)^47^	Yes
5	Yes	Capricorn HIV 1/2 (Capricorn Products, USA)	Yes
6	Yes	C65489M (Meridian Life Science, USA)^47^	Yes
7 (control)	—	NIH-3537 (AIDS Reagent Program, USA)^46^	Yes
8 (control)	Yes	ab89284 (Abcam, UK)	No (anti-GBP5)

For all samples, the proteins listed were diluted in TBS-T buffer containing 2% (w/v) BSA. When HIV p24 is present (column 2), the concentration is 10 nM. When a mouse IgG is present (column 3), the concentration is 200 nM.

We found that the largest phase change was measured for a combination of llama VHH capture ligand and the NIH-3537 detection antibody (labelled immune-complex 3 in Fig. 3b). Figure 3b shows that this combination generated a 5.5° phase shift in response to 4 nM (96ng/ml) p24 within 20 minutes. This corresponds to a five-fold increase in signal generated by the larger mass of the immuno-sandwich complex although additional factors such as surface viscoelastic changes may also contribute to this enhancement. To test the specificity of this signal we ran a control experiments including a sample with no p24 and injecting non-p24-targeting detection antibodies (Fig. 3c). The phase shift measured was significantly smaller than when detecting a p24/anti-p24 immuno-sandwich, indicating that the immuno-sandwich complex gave specific detection of p24.

Next we titrated the concentration of p24 while keeping the anti-p24 antibody fixed and in excess (200 nM − 30 µg/ml). Testing three independent biochips per concentration, a linear relationship was observed in the range 2–40 nM (48–960ng/ml) p24 (Fig. 3d-e) followed by plateauing of the signal - closely fitting a Langmuir adsorption isotherm model (Fig. 3d solid black line fit, *R* ^2^ *=* 0.*96*). This demonstrates the ability of our biosensor to detect HIV p24 proteins down to low nanomolar concentrations in the presence of other background proteins at one million fold higher concentrations (2% w/v or 20 mg/ml BSA). The LDC of p24 was found to be 2 nM (48ng/ml), however, this remains above the clinically relevant concentrations of p24 measured using 4^th^ generation HIV assay laboratory systems (1–1000 pg/ml or 0.04 to 41.7 pM^48^). For example, the bioMérieux VIDAS measures 3–400 pg/ml (0.13–16.7pM) of p24 (manufacturer’s information). A benchmarking study using a quartz crystal microbalance was used to corroborate the p24-VHH assay (Figure S2 in Supplementary Information) and suggest that with identical surface chemistries, the SAW biosensor offers a 20-fold signal enhancement.

### Testing clinical patient plasma samples for HIV

Our results show that SAW biosensors have the ability to rapidly detect biomarkers of HIV with high reproducibility down to low nanomolar (ng/ml) concentrations using model samples in buffer with 2% w/v BSA. As a next step towards the proof of concept of their clinical utility, we tested their performance with anonymised patient samples in partnership with University College London Hospital (UCLH). The challenge of testing patient samples is that blood plasma contains a complex background of serum albumins, globulins, fibrinogen, glucose, clotting factors, hormones, and electrolytes, which are typically present at a million-fold higher concentrations than HIV biomarkers. The high protein concentration of plasma, typically 70 mg/ml, also has a significantly higher viscosity than buffer (1.5 to 1.8 times more viscous^49^). Therefore, it is essential to differentiate the contribution of the *specific* biomarker binding to the surface from *non-specific* background contributions e.g. sample viscosity, to avoid the risk of false positives and false negatives.

**Figure 4:**
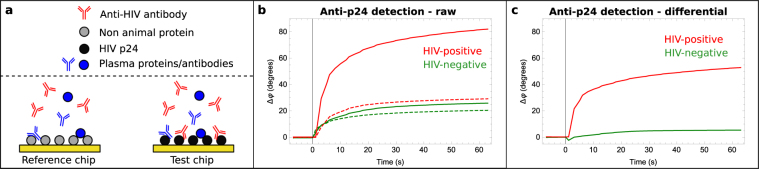
Detection of anti-HIV antibodies in a plasma sample from a patient with HIV (**a**) Schematic of the reference chip functionalized with non-animal protein (NAP), and test chip functionalised with recombinant HIV protein corresponding to the biomarker (HIV p24). Non-specific binding of various plasma proteins and antibodies occurs on both chips, but the biomarker (anti-p24) only binds specifically to the test chip. (**b**) Anti-p24 detection. Phase shift plotted as a function of time for two different samples (one HIV-positive sample in red and one HIV-negative sample in green). Dashed lines represent the reference chips, and the solid lines the test chips. Sample injected at t = 0. (**c**) Differential response of test and reference chips for anti-p24 detection in patient samples. The differential test readout represents the change in phase shift due the specific binding of the biomarker to the SAW biochip, and removes the effect of other non-specific perturbations such as the difference in temperature or viscosity between buffer and plasma. Sample injected at t = 0.

To achieve this, we developed a multiplexed biochip assay involving the detection of HIV anti-p24 antibodies on ‘test’ biochips (coated with p24) and reference biochips (coated with an anti-adsorptive protein, non-animal protein, which has no affinity for either of the HIV biomarkers tested) to account for non-specific signals such as viscosity and temperature (Fig. 4a). The biochips were incubated in buffer for 30 seconds to record a stable baseline. Two patient blood plasma samples were then tested: one HIV positive sample and one negative for anti-HIV antibodies. The phase shifts of both the reference and the test biochips are plotted against time in Fig. 4b. Upon addition of the HIV-positive patient samples, the phase shift of the test biochip (solid line) rapidly increases within 10 seconds to 56° while the reference biochip (dashed line) increases to 20° and effectively plateaus at this level. We remove the non-specific signals that can arise from known differences in viscosity and temperature between buffer and human plasma by taking a differential measurement (test minus reference for each sample). The differential signal shown in Fig. 4c, shows the specific binding of anti-p24 antibodies to p24-coated test chips with a 36° within just 10 seconds (red trace Fig. 4c). By contrast, the differential signal from a healthy volunteer HIV-negative sample gave only a 1° phase shift after 10 seconds.

## Discussion

Our findings show how SAW-based diagnostic devices can be used to rapidly detect biomarkers of HIV infection in model and patient samples with high sensitivity. The microelectromechanical device, comprising a disposable biochip and a small control box which in this study was powered by a laptop, benefits from direct detection of biomarkers on a disposable quartz biochip without the need for any labelling, amplification or wash steps.

We are not aware of any test on the market that can produce such a rapid result. Our findings demonstrate that these SAW devices have the ability to meet the 5 minute time-to-result requirement of an ‘ideal’ HIV self-test outlined by FIND^37^. This proof of concept study is focused on HIV but our platform technology can be applied to a range of different infectious diseases and other non-communicable diseases. The speed of the tests will help to support patients and front-line health-workers by widening access to testing outside of hospital settings, and cut waiting times to give results on the spot. This reduces the need for multiple visits to a clinic to receive a diagnostic result, increases the likelihood that the patient will receive the correct treatment - reducing dependence on presumptive treatments - and reduces the risk that the patient’s health will deteriorate before a correct diagnosis is made. The WHO strongly recommends early testing of infants as a key to initiate treatment faster^50^. Early diagnosis is also known to significantly reduce the risk of onwards transmission by people unaware of their infection^25^ and could be used in a variety of point-of-care settings - from an emergency room to a local doctor’s office, pharmacist, community outreach setting, a rural clinic in a developing country and in the home.

Our findings also highlight the importance of reference sensors and differential measurements. Herein we used individual chips to acquire test and differential measurements but research is underway to engineer a multichannel biochip where up to 4 reagents can be tested simultaneously with a single sample and deployed in a larger pilot clinical study to assess the clinical sensitivity and specificity and time to result of our technology in real world settings (Gray, E. R. *et al.* Manuscript in preparation. (2017)). We show that the platform technology can also be tailored to detect p24, via an immuno-sandwich complex. Further work to understand the fundamental origin of the SAW signal is underway^51^. These developments aim to enable detection of low level of p24 associated with early infections, where a person may unknowingly be 26 times more infectious than during later asymptomatic infection^52^. Indeed, a recent study in North America has shown that early infection accounted for approximately half of all onward transmissions^53^.

Despite these promising results, there are a number of limitations of our current work which we hope to address in future. (i) To date, all the laboratory prototype devices, schematised in Fig. 1a, still require manual pipetting in two steps (buffer stabilisation followed by the addition of sample. The goal is to have a single sample addition step, using the smartphone connected prototype device shown in Fig. 1b. (ii) The biochips used in this work were functionalised manually and this could lead to variations between biochips. In the future, a semi-automated protocol (such as ink-jet printing) will be adopted for biochip functionalisation. (iii) The laboratory prototype has two sets of two different individual biochips (see Fig. 1a) which is necessary to acquire differential measurements in order to compensate for non-specific signals (due to temperature or sample viscosity). However, this set up is bulky and variations between biochips could lead to potential errors. The next generation of biochips have an *in-situ* reference channel coated with a non-specific capture ligand, overcoming the need for multiple chips and reducing the volume of sample required. SAW biochips featuring multiple delay lines have been designed, and work is underway to demonstrate their ability to be used for referencing and multiplexed detection of various biomarkers. Examples include co-infections such as HIV and *Mycobacterium tuberculosis* or syphilis, using a single SAW biochip. (iv) Our current study uses frozen plasma samples and the next step is to use fresh whole blood samples. (v) The analysis of two clinical samples here gives proof of concept. A much larger number of clinical samples are needed to determine clinical sensitivity and specificity. (vi) Future work will investigate the ability of the device to detect different strains of HIV, for example, testing diversity panels representing viruses found worldwide, with controls for other blood-borne viruses such as Hepatitis. (vii) Here we have shown that a SAW biosensor has the required sensitivity needed to detect HIV antibodies in a HIV positive patient sample, however, the detection p24 was not yet within the clinically relevant range. More work is needed to improve the sensitivity to reach pg/ml levels, and subsequently test for p24 in patient samples.

To close, our findings and underlying concepts open up a new field of mass manufacturable, ultra-fast smartphone-enabled consumer diagnostics. True health and economic benefits will be realised when secure on-line patient pathways^54^ are built to link patients to care in developed and developing countries to empower millions of people at risk of HIV, in much the same way that glucose-tests are empowering people with diabetes to manage their own health at home. In future, geo-located information from mobile devices could also be used to support more timely public health responses to emerging infections such as Ebola or Zika viruses.

## Methods

The laboratory prototype SAW biosensor (Fig. 1a) has three components. Firstly, a pocket-sized control box (14 × 10 × 4 cm^3^). Secondly, the disposable SAW biochip (25 × 7× 2 mm^3^). Up to four biochips can be connected in parallel to the control box, allowing for multiplex detection of different biomarkers and control measurements. The control box sends and receives an analogue signal to and from the SAW biochips, and converts it to a digital signal. The third component is the smartphone (or laptop) and associated software, which receives the digital signal from the control box, analyses, displays and then can wirelessly transmit results to healthcare databases.

The SH-SAW biochip was designed using a quartz crystal (36°Y-cut 90°X-propagation). The gold interdigitated transducers (IDT) were evaporated onto the crystal and consisted of 80 finger pairs with an aperture of 1mm, exciting a SH-SAW with a wavelength of 20 µm at a frequency of 251.5 MHz. A 2 nm thin film of titanium was evaporated in between the IDTs, followed by the evaporation of a 90 nm thin film of gold to form the sensing area. The IDTs are protected from liquids by a glass lid and epoxy walls, which are constructed using a photolithography technique as described by Kogai *et al*.^35^.

The gold surface of the SAW biochips was cleaned by incubation in a 2% (volume/volume) solution of Hellmanex III (Hellma Analytics, UK) for 20 minutes, then thoroughly washed with deionised water. It was then functionalised with the relevant capture protein using two successive 30 minute incubations: a first incubation in a solution of Dithiobis[succinimidyl propionate] (DSP - Thermo Scientific Pierce) dissolved in dimethyl sulfoxide (DMSO, Fischer Scientific, UK) at a concentration of 4 mg/ml, followed by incubation in a solution of the capture protein dispersed in phosphate buffer saline (PBS, Sigma-Aldrich, pH 7.4) at 100 µg/ml. The gold surface was washed with DMSO, then PBS in between the two incubations, and with PBS after the second incubation to wash away any unbound protein. On contact with the gold surface, the disulfide bond of DSP cleaves to yield two identical thiols^55^ with an amine-reactive N-hydroxysuccinimide (NHS) ester end, which then reacts during the second incubation with any primary amine available on the capture protein to form a stable amide bond^56^.

Following the functionalisation, the surface was blocked using two successive incubations. First for 15 minutes in a solution of tris buffer saline-tween buffer (TBS-T, pH 7.6 with 0.05% Tween 20, CalbioChem, UK) to block the unreacted DSP molecules via a similar reaction with a primary amine. Secondly in a solution of bovine serum albumin (BSA 2% w/v, Sigma-Aldrich) in TBS-T to reduce unspecific binding during detection of the target protein. The surface was then left incubated in a solution of BSA in TBS-T.

The protocol for testing all samples, including patient plasma samples, involved incubating 20 ul TBS-T buffer on the surface of functionalised SAW biochips for a period of 30 seconds up to 2.5 minutes to establish a stable baseline signal. The buffer was then removed from the surface of the sensor, and replaced with a 20 µl drop of sample, using a micropipette. No further sample handling was needed until the end of the measurement.

Differential measurements were taken by running a test biochip and a reference biochip in parallel: Δφ(differential) = Δφ(test) − Δφ(reference). The reference biochip was functionalised with a protein with no affinity for the analyte (in most cases non-animal protein, G-Biosciences).

The antibodies used were from the following sources: BC1071 (Aalto Bio Reagents, UK), NIH-3537 (AIDS Reagent Program, USA)^46^, NBS500–473 (Novus Biologicals, UK)^47^, Capricorn HIV 1/2 (Capricorn Products, USA), C65489M (Meridian Life Science, USA)^47^, ab89284 (Abcam, UK). The recombinant p24 was obtained from Aalto Bioreagents, UK and is based on GenBank MI5654.

The XPS measurements were conducted at the NEXUS Lab, Newcastle University, UK. The SAW biochips were cleaned using the same protocol as described above. The data were fitted and analysed using CasaXPS software.

The QCM sensors (QSX 301, Biolin Scientific, UK) were cleaned and functionalised with DSP offline, using the same protocol as the one used for the SAW biochips, then inserted into the system (Q-sense E4, Biolin Scientific, UK) to be functionalised with the relevant protein.

The HIV-positive sample was anonymized and had clearance for discard from the UCLH diagnostic laboratory. They were collected as part of the ICONIC project, approved by the Ethical Committee NRES Committee London - Surrey Borders HRA, Research Ethics Committee (REC) London Centre Study title: InfeCtion respONse through vIrus genomiCs (ICONIC) REC reference: 13/LO/1303 HIV viral load was over 5,000 c/mL. Aside from the viral load, no further eligibility criteria were imposed. No clinical measurement of anti-HIV antibody quantitative levels is made by the hospital. The HIV-negative sample was taken after informed consent was obtained from a healthy volunteer from UCL staff. All samples were collected in Ethylenediaminetetraacetic acid BD vacutainers, centrifuged at 3000xg for 20 minutes to separate fractions and plasma, aliquoted and stored at −80 °C until use. All methods were performed in accordance with relevant guidelines and regulations.

The datasets generated during and/or analysed during the current study are available from the corresponding author on reasonable request.

### Ethics statement

The University College London Hospital Research Clinical Microbiology Department reviewed and exempted the HIV-positive samples used in this study from ethics review because it was an assay development, and waived the need for consent due to the fact the patient material used was fully anonymised. HIV-negative samples were obtained from UCL staff and students who gave full informed consent. The study was reviewed by UCL Ethics Board and given study number 6109/001.

### Disclaimer

This article/paper/report presents independent research funded by the National Institute for Health Research (NIHR). The views expressed are those of the author(s) and not necessarily those of the NHS, the NIHR or the UK Department of Health.

## Electronic supplementary material

Supplementary Information
