# Supplementary material for: Towards an ultra-rapid smartphone- connected test for infectious diseases
Source: Sci Rep. 2017 Sep 20;7:11971. doi: 10.1038/s41598-017-11887-6 (PMC5607310; doi:10.1038/s41598-017-11887-6)
Supplement: Supplementary file 1 — Supplementary Information [file 41598_2017_11887_MOESM1_ESM.pdf]

Towards an ultra-rapid smartphone connected test for infectious diseases

Valérian Turbé[1,†], Eleanor R. Gray[1,2,†], Victoria E. Lawson[3], Eleni Nastouli[4], Jennifer C. Brookes[1], Robin A. Weiss[2], Deenan Pillay[2,5], Vincent C. Emery[6], C. Theo Verrips[7], Hiromi Yatsuda[8], Dale Athey[3,*], Rachel A. McKendry[1,*]

[1]London Centre for Nanotechnology, Division of Medicine and Department of Physics and Astronomy, University College London, 17–19 Gordon Street, London WC1H 0AH, UK

[2]Division of Infection and Immunity, University College London, London WC1E 6BT, UK

[3]OJ-Bio, International Centre for Life, Times Square, Newcastle-upon-Tyne, NE1 4EP, UK

[4]Department of Clinical Microbiology and Virology, University College London NHS Foundation Trust, London W1T 4EU, UK

[5]Wellcome Trust Africa Centre for Health and Population Sciences, University of KwaZulu Natal, Mtubatuba, South Africa

[6]Department of Microbial and Cellular Sciences, University of Surrey, Guildford, Surrey, GU2 7TE, UK

[7]QVQ Holding B.V., Utrecht, The Netherlands

[8]Japan Radio Co. Ltd., Saitama 356-8510, Japan

[†]These authors contributed equally to this work.

***Corresponding authors: r.a.mckendry@ucl.ac.uk & dale.athey@oj-bio.com**

A

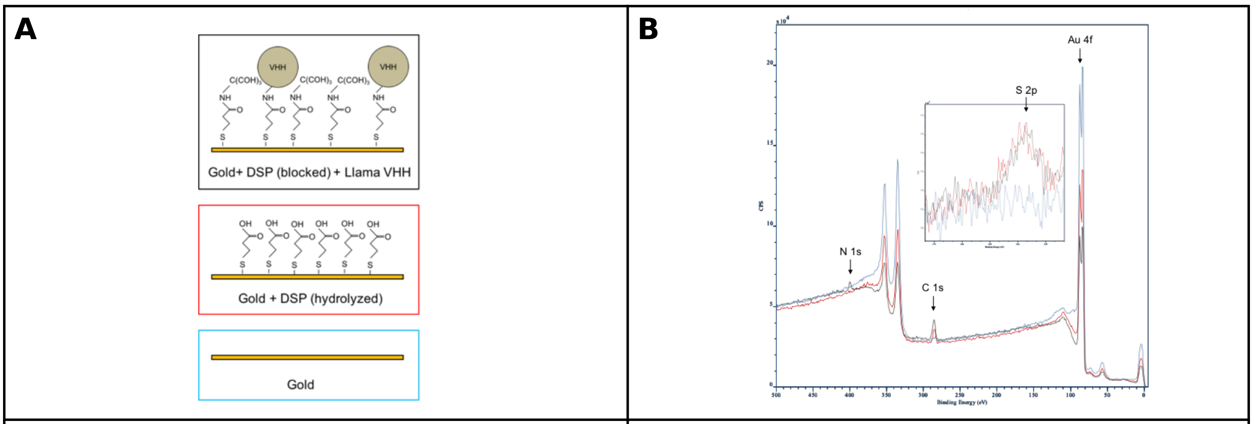

C

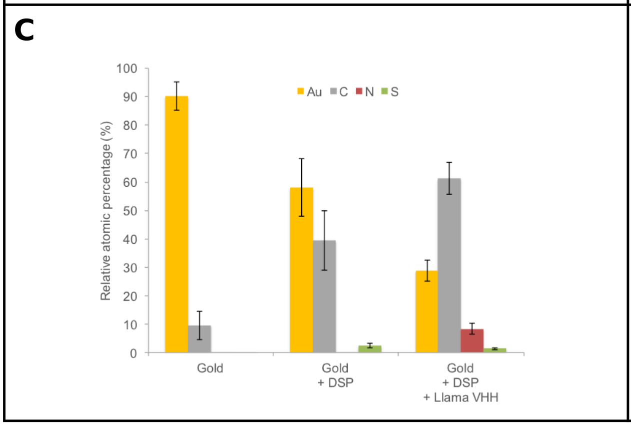

D

Element	XPS	Calculation
C	86.1 ± 10.5	76.7
N	11.8 ± 2.8	18.6
S	2.0 ± 0.6	4.6

Figure S1. XPS characterisation of SAW biochips functionalisation

(a) The surface of SAW biochips was analysed with XPS at different stages of the process: bare gold surface, after DSP functionalisation (and hydrolysis), after llama VHH functionalisation (and blocking of unreacted DSP with Tris).

(b) XPS spectrum survey. CPS (count of electrons per second) as a function of the binding energy. Four elements were selected to analyse the surface composition: Au, C, N and S. One example for each step is plotted. Inset: Zoom on the sulphur peak region.

(c) Relative atomic percentage for the four elements analysed at the different stages of the functionalisation process. A minor amount of impurities on the surface explain the presence of carbon atoms on the bare gold surface. Each bar represents the mean of five independent measurements. Error bars represent the standard deviation of the mean.

(d) The ratios C:N:S measured with XPS compare favourably to the calculated relative percentage of atoms on the surface. The ratios of C:N:S where calculated by finding the planar density of fcc (111) gold (the SAW biochip surface) and a $\sqrt{3}\sqrt{3}R30^{\circ}$ SAM DSP overlayer density and assuming geometrical constraints when the llama VHH is modelled as a $32.2{\times}28.2{\times}48.91\ \text{Å}^3$ cuboid.

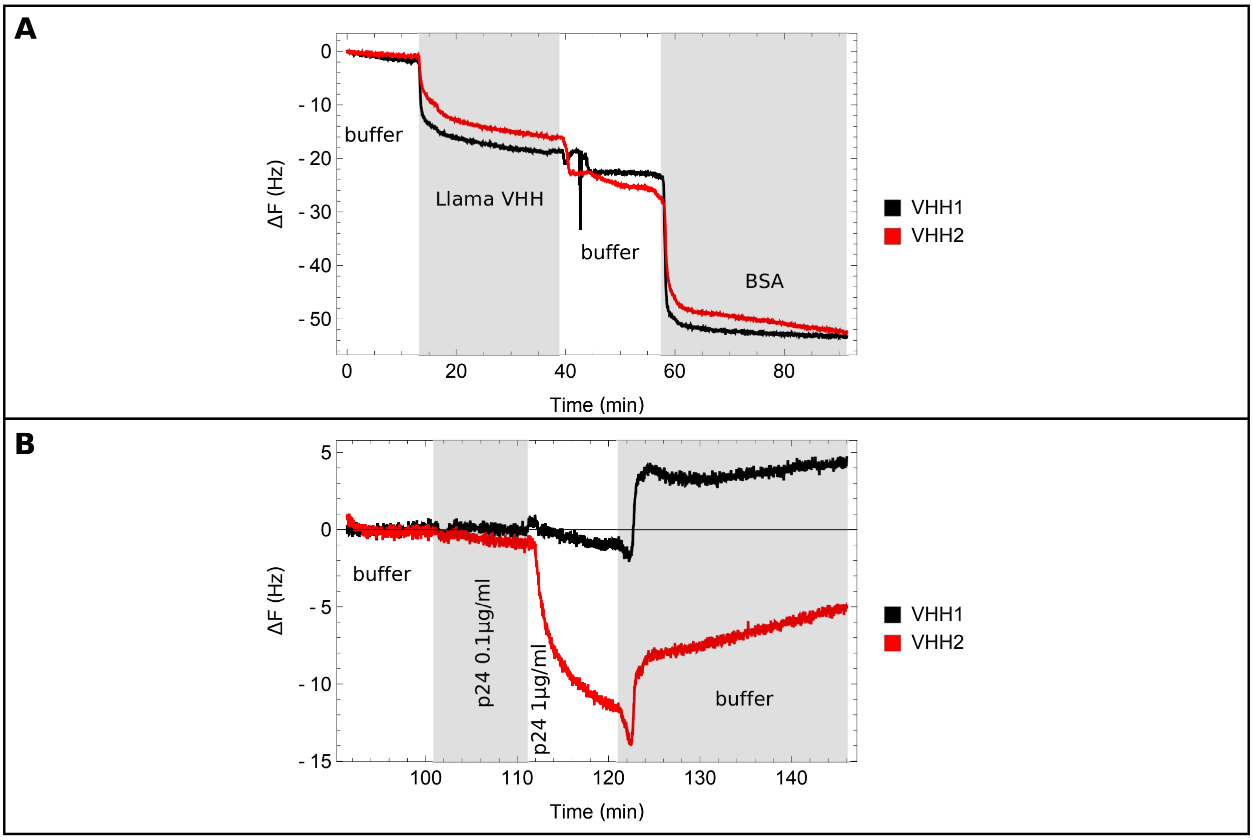

Figure S2. HIV p24 detection assay comparison using QCM

(a) Two gold coated QCM chips were functionalised with llama VHHs of similar molecular weight. VHH1 does not have affinity for p24 and is used as a control, while VHH2 is the one used in the assay described in the study to detect p24. Frequency shift plotted over time as the llama VHHs are injected in the system, and the functionalised surface blocked with BSA (2% w/v in PBS) to avoid non specific binding during the assay.

(b) Frequency shift plotted over time as samples containing a mixture of p24 (increasing concentration, as labelled on graph), anti-p24 (NIH-3537) (200nM) and BSA (2% w/v) in PBS buffer is loaded in the system. Only the chip coated with the anti-p24 llama VHH presents a significant frequency shift upon loading of the sample with a high (1µg/ml) concentration of p24, indicating that this VHH has the ability to capture p24/anti-p24 complexes. Moreover, this same chip does not show any significant frequency shift upon addition of the previous less concentrated sample, despite the presence of BSA and anti-p24 antibodies at high concentrations. This indicate the ability of the anti-p24 VHH to specifically bind to p24.
